# Cohort update: waves 2 and 3 of the global flourishing study

**DOI:** 10.1007/s10654-026-01440-z

**Published:** 2026-07-16

**Authors:** R. Noah Padgett, Richard G. Cowden, Ying Han, Zacc Ritter, Rajesh Srinivasan, Byron R. Johnson, Tyler J. VanderWeele

**Affiliations:** 1 Department of Epidemiology, Harvard T. H. Chan School of Public Health, Boston, MA USA; 2 Human Flourishing Program, Harvard University, Cambridge, MA USA; 3 Gallup, Inc., Ellicott City, MD USA; 4 Gallup, Inc., San Mateo, CA USA; 5 Gallup, Inc., Princeton, NJ USA; 6 Institute for Studies of Religion, Department of Sociology, Institute for Global Human Flourishing, Baylor University, Waco, TX USA; 7 Departments of Epidemiology and Biostatistics, Harvard T.H. Chan School of Public Health, Boston, MA USA

**Keywords:** Global flourishing study, Cross-cultural, Longitudinal, Methodology, Survey sampling

## Abstract

The global flourishing study is a longitudinal panel study designed to advance research on human flourishing across culturally and geographically diverse contexts. wave 1 included more than 200,000 adults from 21 countries and one territory, recruited to be approximately nationally representative of adult (18 +) populations. The initial cohort profile detailed the wave 1 sampling design and implementation. In this cohort profile update, we describe major developments since the original profile was published, including a mid-year retention survey, completion of wave 2 data collection, and inclusion of mainland China data. We summarize attrition patterns across countries, methodological refinements, and potential research applications. These include high variation in retention rates across countries, ranging from 90% in mainland China to 23.5% in Hong Kong, with an overall retention rate of 62%. The overall retention rate at wave 3 overall retention rate of 53.6%. Retention rates for Wave 3 range from 85.3% in mainland China to 12.9% in Hong Kong. The follow-up strategy of recontacting all Wave 1 participants led to the inclusion of ~ 14,000 who did not respond in Wave 2 but did respond in Wave 3. Key strengths of the global flourishing study include its multinational scope, longitudinal design, representative sampling, and open data access, while limitations include reliance on self-reports and variability in some aspects of the methodology across samples (e.g., differences in timing of data collection and weighting approaches). Key limitations of the global flourishing study include the variation and sometimes high attrition rates across some countries, heterogeneity in the length of time between waves, and potential survey mode effects. Beyond a continued commitment to open science, future directions of the global flourishing study include expanding to subsequent waves and the potential addition of new countries and item modules.

**Supplementary Information:**

The online version contains supplementary material available at https://doi.org/10.1007/s10654-026-01440-z.

## Background

The idea of human flourishing has deep historical roots [1]. Although there are different perspectives on how to appropriately conceptualize, measure, and promote human flourishing (for discussion, see [2, 3]), the construct has been defined as “a state in which all aspects of a person’s life are good, including the contexts in which that person lives” p. 8148 [4]. On this view, human flourishing is a multidimensional state that is shaped by the nested socioecological contexts in which people are embedded. The global flourishing study (GFS) draws on this fractal conceptual understanding of human flourishing to advance a large international effort seeking to examine the distribution and determinants of human flourishing in various parts of the world [5, 6]. The study responds to long-standing critiques that research on well-being has been disproportionately concentrated in WEIRD (Western, educated, industrialized, rich, democratic) contexts, limiting generalizability [7–9]. By recruiting nationally representative samples across 22 countries and one territory (hereafter referred collectively as countries for brevity), and by following participants longitudinally, the GFS offers a unique opportunity to broaden the scope of well-being research into a more inclusive and globally representative scientific discipline [10, 11].

Important progress toward this end has already been made since the first wave of GFS data was released (in February 2023) following an open science protocol in partnership with the Center for Open Science. In roughly 18 months since this first data release, more than 70 peer-reviewed scientific articles have been published (see, for example, the ‘global flourishing study—Wave I’ special collection at https://www.nature.com/collections/eaeicjffaf). Many of the published results focus on the distribution and potential childhood predictors of well-being indicators (e.g., life balance, forgivingness, suffering) that have seldom (or never) been examined in nationally representative samples from numerous different cultures and contexts around the world in a single study [12–15].

Now that Wave 2 data collection has been completed and the first two waves of longitudinal panel data have been released, the menu of research questions and analytic options that are possible with the GFS data has expanded exponentially. To support the use of the GFS data moving forward, this cohort update builds on the original cohort profile for Wave 1 [16] by providing information on developments since that publication, including retention and Wave 2 data collection, new dataset releases, methodological updates, and emerging use of the data based on the technical reports for the GFS [16, 17].

## What’s new since the original profile

### Inclusion of mainland China

Although mainland China was not included in the initial Wave 1 release due to fieldwork delays, data collection for waves 1 and 2 was completed in time for incorporation into the second data release in March 2025. The inclusion of China expands the geographic coverage of the GFS to encompass one of the world’s largest populations, increasing coverage of the GFS from approximately half of the global population in the first data release to roughly two-thirds of the global population in the second data release [16, 17]. A detailed description of the sampling design and participant recruitment for the remaining countries is available in the Wave 1 cohort profile paper [16], and a brief overview is provided in our online supplement, with a list of all countries and sampling designs provided in Table A1.

### Mid-year retention survey

Gallup Inc. intended to administer a short mid-year survey between the annual Wave 1 and Wave 2 surveys, with the primary objectives of reminding respondents about their participation in the study and updating each respondent’s contact information for the Wave 2 annual survey [12]. A secondary objective was to administer a limited set of substantive items not included in the annual survey to increase the breadth of constructs covered in the GFS survey while reinforcing engagement with the study to reduce the possibility of attrition. This included questions on social media use, experience of nature, food insecurity, engagement with the arts, experiences of beauty, achievement, engagement, and questions concerning the extent to which various flourishing domains were valued by the participant (see Online Supplement).

There was some variation in how the mid-year survey was administered. A separate mid-year survey was not administered in China, Hong Kong, and Japan due to prohibitive costs; it was also not used in Sweden and the United States because retention rates were already high. In Argentina, Brazil, Germany, Mexico, Spain, and the United Kingdom, the non-probability samples did not complete a separate mid-year survey, but the probability samples did. Finally, the face-to-face sample in Nigeria did not complete a separate mid-year survey. Respondents who did not complete a separate mid-year survey were presented with the mid-year survey questions during Wave 2 annual survey data collection to ensure all respondents had an opportunity to answer these items. Respondents in a total of 11 countries were administered a mid-year survey that was entirely separate from the annual Wave 2 survey to the full sample. The second data release includes an indicator variable to identify whether a respondent belongs to a sample invited to complete a separate mid-year survey or to a sample that completed the intended mid-year survey questions jointly with the Wave 2 annual survey [18].

**Fig. 1:**
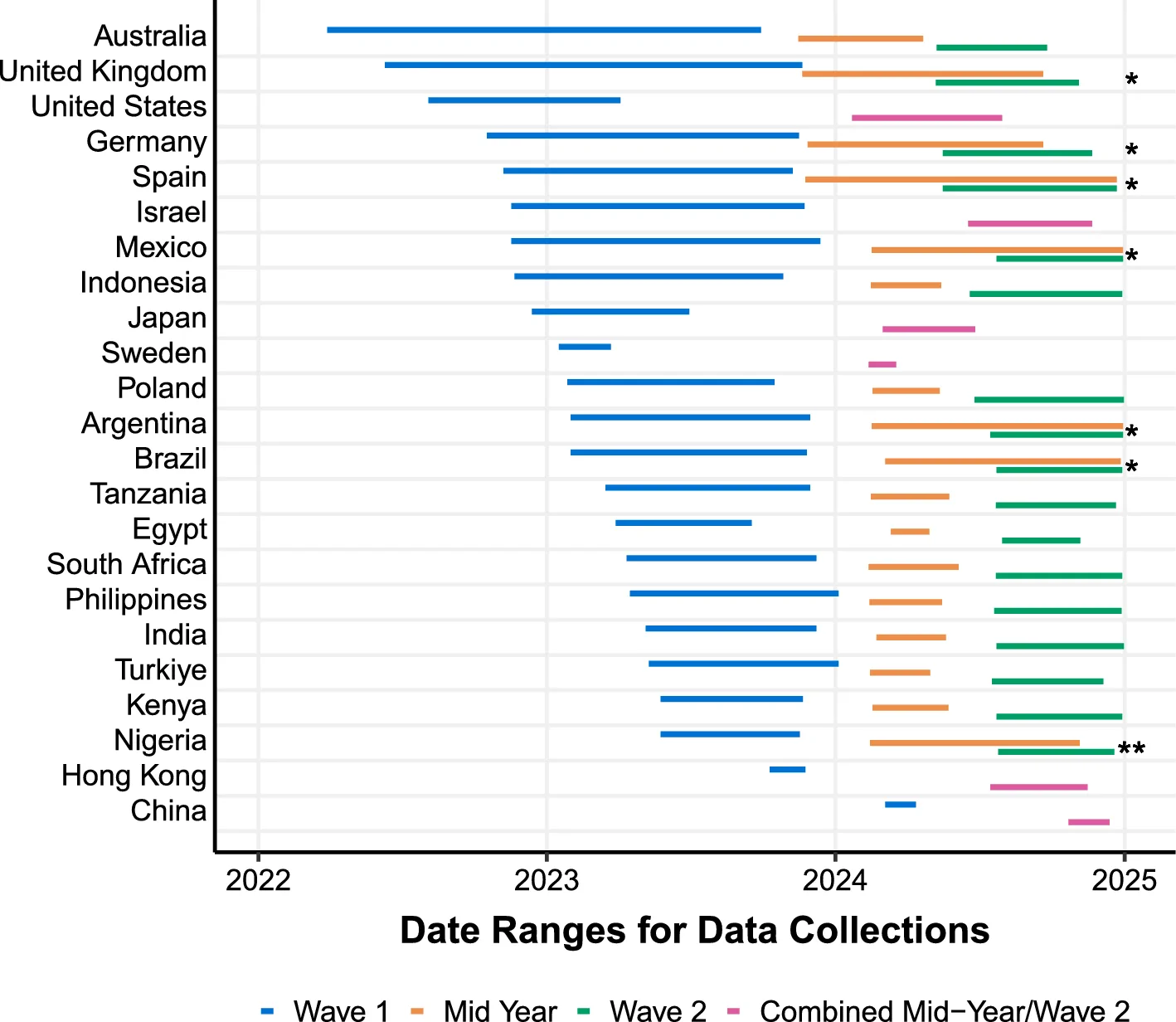
Date ranges of data collection for the annual wave 1, Mid-year retention, and annual wave 2 surveys of the global flourishing study. *Note* *Mid-year data collection occurred concurrently with the Annual Wave 2 survey for the non-probability sample within Argentina, Brazil, Germany, Mexico, Spain, and the United Kingdom. **Midy-year data for the face-to-face sample in Nigeria was administered concurrently with the Annual Wave 2 survey. All mid-year data collection in China, Hong Kong, Israel, Japan, Sweden, and the United States occurred concurrently with the Annual Wave 2 survey

For samples that were invited to participate in a separate mid-year survey, there were slight differences in the approach used to recontact respondents based on whether their participation in the Wave 1 Annual Survey was via telephone or online. Five attempts were made to contact panelists who were taking the telephone survey. Panelists taking the survey via the web received an initial invitation followed by five reminders to participate in the online survey across all channels through which they had consented to receive communications, including email, SMS, and WhatsApp. The dates for the mid-year retention survey across countries are shown in Fig. 1 and provided in Table C1.

### Wave 2 data collection

Wave 2 data collection began at least six months after Wave 1 completion within the country (see Table C2 for a summary of the time elapsed between data collections by country). To eliminate analytical challenges posed by potential within-subject mode effects (see Dillman [19] for a discussion of such effects), respondents were not permitted to switch survey methods and completed the Wave 2 Annual Survey using the same mode they used to complete the Wave 1 Annual Survey. For example, a respondent who completed the annual survey by telephone in Wave 1 was required to complete the survey by telephone again in Wave 2.

For respondents taking the annual survey by telephone, five initial attempts were made to contact panelists using the primary telephone number on record. If the respondent did not complete the survey, an additional five attempts were made using the primary telephone number and an alternate telephone number when available for a maximum of 10 attempts at contact. For respondents taking the annual survey by web, an initial invitation followed by five reminders to participate in the online survey across all channels through which they consented to receive communications, including email, SMS, and WhatsApp. If the respondent did not complete the survey after these outreach attempts, the local field partner made three attempts to contact panelists. In the case of successful contact, the interviewer reminded the respondent about their participation in the study, updated contact information as needed, and asked them to complete the web survey [17].

The overall retention rate from Wave 1 to 2 is 62.0%, with variation across countries. Retention rates were lowest in Hong Kong at 23.5% and Brazil at 32.4%. Retention rates were highest in China at 90.5% and in the United States at 84.2%. Table 1 provides the sample sizes and retention rates across countries. Eleven of the 22 countries and one territory had a retention rate above 60% and 17 of the countries had a retention rate above 40%. Another key factor concerning retention rate is the mode of participation, with retention rates of 64.6% for web-based completion and 55.5% for phone-based completion of the Wave 2 Annual Survey (see Table C3 for breakdown of retention rates by administration mode and country). The widest gap in retention rates by mode occurred for Israel, where web-based completion was 26.6% while phone-based completion was 81.8%. These patterns highlight some of the challenges associated with maintaining participation over time in large-scale international panels and should be taken into consideration when interpreting results using the longitudinal GFS data.

**Table 1:** Retention rates across mid-year retention and annual wave 2 data collections

	Annual wave 1	Mid-year		Annual wave 2	
Country	N	N	%-Retained	N	%-Retained
Overall	207,919	131,487	63.2	128,868	62.0
China*	5,022	4,544	90.5	4,544	90.5
United States*	38,312	32,192	84.0	32,245	84.2
Sweden*	15,068	11,570	76.8	11,609	77.0
Japan*	20,543	13,886	67.6	13,972	68.0
Israel*	3,669	2,476	67.5	2,490	67.9
Kenya	11,389	9,115	80.0	7,698	67.6
United Kingdom	5,368	3,354	62.5	3,619	67.4
Australia	3,844	2,533	65.9	2,582	67.2
Egypt	4,729	3,386	71.6	3,040	64.3
Poland	10,389	4,316	41.5	6,478	62.4
Tanzania	9,075	6,577	72.5	5,583	61.5
Germany	9,506	4,163	43.8	5,529	58.2
Philippines	5,292	3,448	65.2	2,682	50.7
India	12,765	8,206	64.3	6,374	49.9
Spain	6,290	2,215	35.2	2,924	46.5
Nigeria	6,827	4,943	72.4	3,146	46.1
Argentina	6,724	2,728	40.6	2,932	43.6
Mexico	5,776	2,126	36.8	2,278	39.4
Indonesia	6,992	2.675	38.3	2,684	38.4
South Africa	2,651	1.625	61.3	978	36.9
Turkey	1,473	659	44.7	500	33.9
Brazil	13,203	4,044	30.6	4,274	32.4
Hong Kong*	3,012	707	23.5	707	23.5

*Wave 2 Annual Survey and Mid-year Retention Survey administered simultaneously in these countries so the sample size and retention rates are approximately equal except for a small percentage of cases that did not complete the retention survey after completing the annual survey. An expanded version of this table is provided in our supplemental material (Table C3) breaking down retention rates by country and mode of administration

Retention was relatively low for the Central and South American countries: Brazil at 32.4%, Argentina at 43.6%, and Mexico and 39.4%. While the overall retention rate for the study was quite good (above 60%), the lower retention rates in these countries, where flourishing and subjective well-being are less frequently studied, may be concerning. One reason the retention rates might be lower in Argentina is the misalignment of the Argentine sample in the GFS with respect to educational characteristics. Vanney and colleagues [20] highlighted how the distribution of educational attainment in the GFS, noting that “Compared with the 2022 population [Argentine] census data, the segment of the population with the lowest level of education is notably over-represented [in the GFS] and the segment with the highest level of education is considerably sub-represented in the sample, introducing a possible bias in the responses.” (p.19, with brackets added for clarity). Less educated participants are also more likely not to participate in Wave 2 of the study, so the lack of alignment on education could be inducing higher rates of attrition.

#### Effect of attrition

Attrition has different effects on the validity of inference depending on the analyses being conducted. For cross-sectional analyses of Wave 2 data within each country, attrition is generally less of a concern than for longitudinal analyses because Wave 2 can be treated as its own sample. However, inference still depends on how well poststratification and raking variables capture factors driving nonresponse, and severe attrition can produce unstable weights and reduce precision. For cross-country comparisons at Wave 2, differential attrition across countries can affect comparability if nonresponse mechanisms and reweighting effectiveness differ by country. Attrition will typically be most consequential for longitudinal analyses, such as when examining Wave 1 predicts of Wave 2 outcomes, because selection into Wave 2 might depend on Wave 1 characteristics (observed and unobserved) that have the potential to confound effect estimates.

#### Heterogeneous lags

The length of time between dates of data collection varies within and between countries (see Table C2). The average lag between Waves 1 and 2 was 417.5 days (SD = 98.2 days) across all study participants. This varied considerably across countries, with the shortest lag (excluding mainland China, where data collection for both waves occurred in the same calendar year) being in Hong Kong, with an average lag of 301.9 days (SD = 19.8), and the longest lag being in Australia, with an average lag of 565.9 days (SD = 146.0). The average within-country lag varied by approximately 70 days across countries.

The heterogeneity in data collection dates has some potential consequences for inference from analyses using multiple waves of data. First, variance in the length of time between data collection points could lead to estimates of association that vary across countries, not because the associations differ, but because the length of time allowed for sufficient change in the outcome at Wave 2 to be detected. For example, overall life satisfaction or meaning in life are considered relatively stable over relatively short time periods of 1–2 years [21, 22], thus changes may not be detectable in countries with shorter lags on average. Secondly, the inverse is also plausible, where the length of time is too long in some countries for the change to still be detectable, either because the construct changes too rapidly or might be cyclical (e.g., frequency of exercising). Third, the heterogeneity in lag could make within-country reasoning about associations between variables across waves potentially confounded by time-dependent confounding. For example, societal events may occur within a wave of data collection that separates participants into “pre-event” and “post-event” groups, such as for participants in Israel, where some completed Wave 1 before the Israel-Hamas conflict began on October 7, 2023 while others completed it after. Depending on the goals of one’s analyses, it might be preferable to incorporate the “lag” as a moderating variable of potential associations to try to account for the differential lag within a country [23]. We caution researchers using these data to think critically about whether the outcomes and associations of interest are subject to time-varying effects and what impact differential lag may have on these associations.

One way researchers can probe these effects in their analyses is to conduct sensitivity analyses by stratifying by the lag to create “lag groups” when sample sizes are sufficient. The analyses can then be conducted separately within each lag stratum (e.g., < 300 days, 300–399 days, 400–499 days, 500 + days; or create quantile-based brackets within country) to assess whether the estimates of association within each country are consistent across lags. This is a relatively crude approach that may be useful when there is no a priori reason to believe lag may moderate any associations. The more rigorous incorporation of the moderating effect of lag may be similarly useful.

## Weighting methodology

Weighting is used to ensure samples are approximately nationally representative for adult (18 +) population in each country or territory [16, 17]. These weights are intended for use in calculations within a single country and should not, in general, be used for combined analyses across multiple countries due to differences in each country's population size and the observed sample size for each country. Survey weights adjust for unequal probabilities of selection and differential response rates, thereby helping ensure estimates are more representative of the population than the sample. When analyzing survey data, using appropriately calibrated weights is essential to ensure that results accurately reflect the target population within each country.

Weighting for the GFS data is conducted separately for each country to ensure the adjustments account for country-specific variation in data collection procedures. However, the process used to adjust weights is the same across countries, but using the country-specific information and population targets. Separate weights were created to facilitate approximately nationally representative statistics for each of: (1) analyses restricted to participants who participated in a specific wave, (2) analyses restricted to participants participating in multiple waves, and (3) analyses using all empaneled participants regardless of whether they participated in a particular wave. In all such cases, the following process was used to create a weighting variable.

### Weight adjustments

Weighting begins with a base weight that estimates the selection probability of a GFS panel member, which remains the same as the Wave 1 annual weight. A detailed description of the Wave 1 annual weight construction can be found in the GFS Wave 1 Methodology Report [17] and in the accompanying survey documentation [16]. The Wave 1 sampling weights were adjusted using a logistic regression model to predict the probability of participating in Wave 2. The adjustment factor reflects the inverse of the estimated probability of participating. Respondents with a higher probability of participating receive a lower weight, while those with a lower probability receive a higher weight. This adjustment is then multiplied by the Wave 1 sampling weight to correct for the over-representation of individuals more likely to participate and the underrepresentation of those less likely to participate. A more detailed description is provided in our supplemental material (see section B).

### Aligning weights with analysis

The above weighting procedures result in multiple types of weights depending on how nonresponse and attrition are handled. Researchers must choose what type of weight to use that aligns with their analysis. First, if the analysis only uses variables from Wave 1, then using the weight developed using the Wave 1 weighting procedures for inference is optimal (see Padgett and colleagues [16] for more details). Second, if the analysis only uses Wave 2 variables, then the cross-sectional weight developed for separate Wave 2 analyses should be used. This is the attrition-adjusted weight that aligns the Wave 2 complete-case sample with population targets and characteristics [17]. For example, summarizing the average score on the Flourishing Index for each country at Wave 2 can utilize this type of weight. Third, longitudinal analyses can use a weight based on the complete-case sample (participants who completed Wave 1 and Wave 2) or on the full sample (all participants who completed Wave 1 regardless of status at Wave 2). For example, regressing Wave 1 religious service attendance on Wave 2 Flourishing Index scores can use this type of weight. In both types of analyses, researchers will need to address item-level missingness to provide valid population-level inferences. Excluding any cases with valid weight (i.e., listwise deletion) results in the weighted analysis projecting to the incorrect population. A description of each specific weight variable is provided in the online supplemental material.

### Limitations of weights

Above, we discussed how weights were adjusted for nonresponse to create what is known as a nonresponse-adjusted sampling weight, which comes with several caveats for its use. First, the newly created weights assume the model used to construct the adjustment factor is correct. This means that if the logistic regression model omits a key variable related to nonparticipation, the resulting predicted probabilities and weights are biased, leading to biased inferences about the population. Second, the full-sample weights do not adjust for nonparticipation in Wave 2, but missing data may be present if analyzing data from both waves. The valid use of the weights for population-level inferences requires missingness to be accounted for. One potentially valid way to handle the missingness is to use multiple imputation. However, multiple imputation has the same assumption as the logistic regression model, in that it must be correctly specified for valid imputations. In both cases, the general condition is known as missing at random (MAR), meaning that the dataset has enough information to inform the values that are missing. MAR is a difficult assumption to meet. While the GFS dataset has a very broad set of measures that provide some confidence that using all available data to inform missingness may be a good approximation of the missingness model, there is no way to definitively rule out nonrandom missingness in the GFS.

## Attrition and sample characteristics

A crucial question is how aligned the samples are with the population. The claim of analyses being “approximately nationally representative of the adult (18 +) population” depends on this alignment. The Wave 1 empaneled sample was weighted to country-specific targets, resulting in this approximately nationally representative claim. However, in Wave 2 and later waves, this claim has the added wrinkle of attrition, which needs to be incorporated into the adjustment of the weights to ensure the sample remains aligned with the population.

### Sample statistics between waves

To help evaluate alignment across waves, we next break down differences in sample age and percent female by country, and compare unweighted and weighted statistics to illustrate these potential differences. The unweighted statistics provide valuable information about how potentially unrepresentative the sample is if the appropriate weighting adjustments are not used, while the weighted statistics illustrate the demographic characteristics of the sample when analyzed with the appropriate weighting adjustments. Aggregating over all countries, the weighted and unweighted sample statistics for age, gender, marital status, education, employment status, and urbanicity are reported in Table 2. The unweighted statistic shows how the sample gender distribution balances out slightly to roughly equal representation of males and females, a greater percentage of respondents report being married in Wave 2 than in Wave 1, a greater percentage of respondents report completing 16 or more years of education, and a greater percentage of respondents report being retired. After weighting, most differences between waves decrease to less than 3 percentage points. A notable exception occurs for education, which shifts more severely towards more educated participants, with the overall percentage of participants with 16 or more years rising from 21.0% to 25.7% (+ 4.7%).

**Table 2:** Comparison of (un) weighted sample statistics for global flourishing study

	Unweighted			Weighted	
Characteristic	Wave 1 *(N* = *207,919)*	Wave 2 *(N* = *128,868)*		Wave 1 *(N* = *207,919)*	Wave 2 *(N* = *128,868)*
Age of participant					
Mean (SD)	45.8 (17.6)	50.1 (17.5)		45.0 (17.3)	47.3 (17.4)
(Missing)	20 (< 0.1%)	2 (< 0.1%)		20 (< 0.1%)	1 (< 0.1%)
Gender, n (%)					
Male	97,761 (47.0%)	62,823 (48.7%)		100,932 (48.5%)	62,309 (48.4%)
Female	109,331 (52.6%)	65,617 (50.9%)		105,985 (51.0%)	66,020 (51.2%)
Other	406 (0.2%)	238 (0.2%)		602 (0.3%)	349 (0.3%)
(Missing)	421 (0.2%)	190 (0.1%)		397 (0.2%)	189 (0.1%)
Respondent marital status, n (%)					
Single/Never been married	50,885 (24.5%)	26,685 (20.7%)		53,133 (25.6%)	30,394 (23.6%)
Married	112,740 (54.2%)	74,441 (57.8%)		110,905 (53.3%)	71,036 (55.1%)
Separated	5229 (2.5%)	3,227 (2.5%)		5,236 (2.5%)	3,058 (2.4%)
Divorced	12,283 (5.9%)	8,587 (6.7%)		11,770 (5.7%)	8,189 (6.4%)
Widowed	9,323 (4.5%)	6,791 (5.3%)		9984 (4.8%)	6,473 (5.0%)
Domestic partner	15,604 (7.5%)	8,316 (6.5%)		15,058 (7.2%)	8,817 (6.8%)
(Missing)	1,855 (0.9%)	821 (0.6%)		1,830 (0.9%)	901 (0.7%)
Education (years), n (%)					
Up to 8	33,803 (16.3%)	16,572 (12.9%)		45,673 (22.0%)	22,941 (17.8%)
9–15	118,629 (57.1%)	69,954 (54.3%)		118,388 (56.9%)	72,836 (56.5%)
16 or more	55,300 (26.6%)	42,331 (32.8%)		43,709 (21.0%)	33,080 (25.7%)
(Missing)	187 (0.1%)	11 (< 0.0%)		146 (0.1%)	11 (< 0.0%)
Employment status, n (%)					
Employed for an employer	76,004 (36.6%)	48,932 (38.0%)		80,736 (38.8%)	53,831 (41.8%)
Self-employed	35,985 (17.3%)	20,552 (15.9%)		37,672 (18.1%)	20,719 (16.1%)
Retired	34,715 (16.7%)	28,816 (22.4%)		30,225 (14.5%)	22,158 (17.2%)
Student	10,910 (5.2%)	3,888 (3.0%)		10,897 (5.2%)	5,085 (3.9%)
Homemaker	23,128 (11.1%)	11,158 (8.7%)		21,966 (10.6%)	11,304 (8.8%)
Unemployed and looking	17,252 (8.3%)	8,684 (6.7%)		16,952 (8.2%)	8,965 (7.0%)
None of these/Other	9,101 (4.4%)	5,984 (4.6%)		8,675 (4.2%)	5,842 (4.5%)
(Missing)	824 (0.4%)	854 (0.7%)		794 (0.4%)	964 (0.7%)
Urbanicity status, n (%)					
A rural area or on a farm	38,713 (18.6%)	22,859 (17.7%)		38,527 (18.5%)	22,788 (17.7%)
A small town or village	72,276 (34.8%)	44,814 (34.8%)		73,584 (35.4%)	45,791 (35.5%)
A large city	56,749 (27.3%)	33,558 (26.0%)		57,026 (27.4%)	33,524 (26.0%)
A suburb of a large city	38,926 (18.7%)	27,203 (21.1%)		37,712 (18.1%)	26,298 (20.4%)
(Missing)	1,255 (0.6%)	434 (0.3%)		1,067 (0.5%)	467 (0.4%)

Weighted statistics were estimated using the survey package in R. For Wave 1 estimates, the *annual_weight_c1* was used, and for Wave 2 estimates, the *annual_weight_c2* was used; see methodology document for more details [17]

Differences in the sample characteristics between waves are also explored within each country. The weighted and unweighted sample characteristics are provided in Table 3 for respondent age and the percentage of females for each country. For age, we would expect to see an increase of about 1–2 years for most countries (see Table C2 for the average difference in days between survey administrations by country). In contrast to this expectation, we see the unweighted average age of the sample increased by as much as 5.8 years in Mexico. After the weighting adjustments, the average difference in age between Waves 1 and 2 drops to only 0.7 years in Mexico, which might be more suggestive that the Wave 2 sample may be overly representative of older respondents. Additional breakdown of the sample characteristics by marital status, education, employment status, and urbanicity are reported in our supplemental material (see supplemental file “*Sample Statistics by Wave and Country.xlsx*”). In general, these results provide some evidence that, after weighting, the sample is approximately representative of the same population, with some deviations, such as the retained sample being more educated, resulting in a potential misalignment of the target population even after weighting.

**Table 3:** Comparison of (un)weighted ages and percent female estimates across countries for the global flourishing study

	Age of participants, mean (SD)				Percent female			
	Unweighted		Weighted		Unweighted		Weighted	
Country	Wave 1	Wave 2	Wave 1	Wave 2	Wave 1	Wave 2	Wave 1	Wave 2
Argentina	42.4 (15.5)	47.5 (15.3)	42.4 (16.7)	44.3 (16.5)	55.7%	52.3%	52.7%	50.4%
Australia	57.0 (16.8)	60.0 (15.9)	49.9 (18.1)	51.6 (17.7)	50.7%	50.3%	50.5%	50.3%
Brazil	37.8 (13.9)	43.4 (14.1)	41.9 (15.7)	44.1 (16.3)	56.3%	54.8%	51.7%	51.5%
China	44.6 (15.3)	45.2 (15.3)	45.0 (15.2)	45.4 (15.4)	49.2%	49.6%	49.7%	49.4%
Egypt	38.5 (12.6)	40.0 (12.5)	38.0 (13.9)	38.3 (14.1)	58.4%	57.9%	49.4%	48.6%
Germany	48.5 (16.7)	51.9 (16.4)	49.7 (17.2)	50.7 (17.1)	49.7%	49.6%	51.0%	51.5%
Hong Kong	44.3 (13.4)	45.2 (11.7)	48.3 (15.1)	48.7 (14.9)	54.2%	60.0%	53.8%	55.9%
India	37.3 (13.7)	38.3 (13.4)	38.6 (14.9)	39.3 (14.3)	53.6%	49.7%	49.3%	48.5%
Indonesia	37.2 (12.1)	38.3 (11.9)	39.2 (13.7)	39.9 (13.9)	58.4%	56.3%	50.2%	50.0%
Israel	44.0 (17.7)	46.9 (18.0)	44.7 (17.8)	45.6 (18.2)	51.0%	49.6%	51.0%	51.3%
Japan	52.5 (17.0)	56.0 (15.6)	52.5 (17.1)	53.3 (17.0)	52.1%	49.8%	51.6%	52.6%
Kenya	32.5 (12.5)	34.4 (12.4)	36.4 (15.0)	36.8 (14.7)	54.5%	53.1%	51.0%	51.1%
Mexico	38.8 (15.2)	44.4 (15.5)	41.8 (16.2)	42.5 (16.2)	60.1%	56.9%	51.9%	52.1%
Nigeria	31.4 (10.1)	33.5 (10.8)	35.4 (13.6)	36.1 (13.7)	45.1%	43.4%	50.6%	48.9%
Philippines	39.8 (14.5)	41.5 (14.2)	39.3 (15.3)	40.0 (15.3)	69.3%	70.7%	49.9%	51.6%
Poland	44.2 (14.1)	46.3 (14.4)	47.1 (16.5)	47.9 (16.5)	53.6%	54.1%	51.8%	52.4%
South Africa	36.2 (13.0)	37.1 (13.1)	39.5 (15.2)	39.8 (15.0)	58.9%	60.0%	51.2%	51.9%
Spain	43.3 (14.3)	48.1 (13.5)	46.7 (15.6)	47.7 (15.5)	54.3%	53.3%	49.6%	49.9%
Sweden	48.5 (19.0)	52.5 (18.1)	49.4 (18.7)	50.0 (18.4)	48.8%	50.2%	49.7%	50.6%
Tanzania	37.8 (14.3)	40.3 (14.1)	37.1 (15.4)	37.6 (14.9)	55.6%	52.1%	52.6%	51.9%
Turkey	38.5 (13.6)	42.4 (13.4)	42.3 (15.9)	43.2 (16.1)	41.5%	37.8%	48.8%	50.0%
United Kingdom	54.2 (17.5)	57.6 (16.2)	49.4 (17.8)	51.0 (17.1)	49.9%	49.7%	51.9%	51.6%
United States	59.3 (15.5)	60.9 (15.1)	49.4 (17.6)	52.9 (16.5)	48.1%	47.7%	51.1%	51.9%

Weighted statistics were estimated using the survey package in R. For Wave 1 estimates, the *annual_weight_c1* was used, and for Wave 2 estimates, the *annual_weight_c2* was used; see methodology document for more details [14]

### Wave 1 characteristics by retention status

Next, we provide a high-level summary of the differences in Wave 1 characteristics between retained and non-retained participants (see Table 4). Non-retained participants tended to be younger, and a higher percentage of those not retained were female, single, had only up to 8 years of education, were unemployed, and lived in a large city. Additionally, non-retained participants had slightly lower average Secure Flourishing Index scores (mean difference = 0.10, standardized mean difference = 0.06).

**Table 4:** Comparison of demographic characteristics at Wave 1 between retained and non-retained cases (all statistics are unweighted)

Characteristic	Retained *(N* = *128,868)*	Not-retained (*N* = 79,051)	Overall (*N* = 207,919)
Secure flourishing index			
Mean (SD)	7.16 (1.65)	7.06 (1.69)	7.12 (1.67)
(Missing)	3,182 (2.5%)	2,386 (3.0%)	5,568 (2.7%)
Age of participant			
Mean (SD)	49.02 (17.62)	40.54 (16.29)	45.80 (17.61)
(Missing)	10 (< 0.1%)	10 (< 0.1%)	20 (< 0.1%)
Gender, n (%)			
Male	62,823 (48.7%)	34,938 (44.2%)	97,761 (47.0%)
Female	65,617 (50.9%)	43,714 (55.3%)	109,331 (52.6%)
Other	238 (0.2%)	168 (0.2%)	406 (0.2%)
(Missing)	190 (0.1%)	231 (0.3%)	421 (0.2%)
*Respondent marital status, n (%)*			
Single/Never been married	27,961 (21.7%)	22,924 (29.0%)	50,885 (24.5%)
Married	74,144 (57.5%)	38,596 (48.8%)	112,740 (54.2%)
Separated	2,867 (2.2%)	2,362 (3.0%)	5,229 (2.5%)
Divorced	8,525 (6.6%)	3,758 (4.8%)	12,283 (5.9%)
Widowed	6,327 (4.9%)	2,996 (3.8%)	9,323 (4.5%)
Domestic partner	8,233 (6.4%)	7,371 (9.3%)	15,604 (7.5%)
(Missing)	811 (0.6%)	1,044 (1.3%)	1,855 (0.9%)
Education (years), n (%)			
Up to 8	18,321 (14.2%)	15,482 (19.6%)	33,803 (16.3%)
9–15	70,512 (54.7%)	48,117 (60.9%)	118,629 (57.1%)
16 or more	39,909 (31.0%)	15,391 (19.5%)	55,300 (26.6%)
(Missing)	126 (0.1%)	61 (0.1%)	187 (0.1%)
Employment status, n (%)			
Employed for an employer	48,161 (37.4%)	27,843 (35.2%)	76,004 (36.6%)
Self-employed	20,685 (16.1%)	15,300 (19.4%)	35,985 (17.3%)
Retired	27,606 (21.4%)	7,109 (9.0%)	34,715 (16.7%)
Student	5,412 (4.2%)	5,498 (7.0%)	10,910 (5.2%)
Homemaker	12,169 (9.4%)	10,959 (13.9%)	23,128 (11.1%)
Unemployed and looking for a job	8,913 (6.9%)	8,339 (10.5%)	17,252 (8.3%)
None of these/Other	5,665 (4.4%)	3,436 (4.3%)	9,101 (4.4%)
(Missing)	257 (0.2%)	567 (0.7%)	824 (0.4%)
Urbanicity status, n (%)			
A rural area or on a farm	23,453 (18.2%)	15,260 (19.3%)	38,713 (18.6%)
A small town or village	44,519 (34.5%)	27,757 (35.1%)	72,276 (34.8%)
A large city	33,350 (25.9%)	23,399 (29.6%)	56,749 (27.3%)
A suburb of a large city	27,171 (21.1%)	11,755 (14.9%)	38,926 (18.7%)
(Missing)	375 (0.3%)	880 (1.1%)	1,255 (0.6%)

### Sampled population across survey modes

In 16 of the countries included in the GFS, data were collected using a combination of web surveys and telephone interviews [17]. Retention rates were generally higher for participants in the phone interviews (see Table C3). A concern in these data is that there are mode effects associated with participation in the study and with the responses participants provide. A major confounding factor is the baseline differences in the sample between modes. Table 5 provides a breakdown of the distribution of key demographic characteristics by retention status and survey mode in these 16 countries. First, looking at the baseline differences in the characteristics by survey mode highlights how participants using the web survey tend to be older and not married compared to the phone interview participants. The up to 8 years of education bracket accounted for 40.8% of the phone interview participants, while this education bracket only accounted for 10.7% of the web survey participants.

**Table 5:** Differences in sample characteristics between survey modes and retention status—restricted to the 16 countries with phone and web surveys

			Phone interview			Web survey		
Characteristic	Phone N = 61,046	Web N = 64,923	Retained (N = 33,910)	Not-retained (N = 27,136)	d^*^	Retained (N = 35,287)	Not-retained (N = 29,636)	d^*^
Age of participant								
Mean (SD)	38.82 (14.88)	42.41 (16.26)	39.37 (14.94)	38.13 (14.78)	**1.24**	46.04 (16.40)	38.08 (14.98)	**7.96**
(Missing), *n (%)*	10 (< 0.1%)	2 (< 0.1%)	5 (< 0.1%)	5 (< 0.1%)	0.0	1 (< 0.1%)	1 (< 0.1%)	0.0
Gender, n (%)								
Male	27,167 (44.5%)	29,565 (45.5%)	15,784 (46.5%)	11,383 (41.9%)	4.6	16,912 (47.9%)	12,653 (42.7%)	5.2
Female	33,865 (55.5%)	35,017 (53.9%)	18,120 (53.4%)	15,745 (58.0%)	*-* 4.6	18,253 (51.7%)	16,764 (56.6%)	*-* 4.9
Other	13 (0.0%)	118 (0.2%)	5 (< 0.1%)	8 (< 0.1%)	0.0	41 (0.1%)	77 (0.3%)	*-* 0.2
(Missing)	1 (< 0.0%)	223 (0.3%)	1 (< 0.1%)	0 (0%)	0.1	81 (0.2%)	142 (0.5%)	*-* 0.3
Respondent marital status								
Single/Never married	14,599 (23.9%)	20,238 (31.2%)	7,617 (22.5%)	6,982 (25.7%)	*-* 3.2	10,112 (28.7%)	10,126 (34.2%)	*-* 5.5
Married	37,944 (62.2%)	30,693 (47.3%)	21,679 (63.9%)	16,265 (59.9%)	**4.0**	18,885 (53.5%)	11,808 (39.8%)	**13.7**
Separated	1,745 (2.9%)	1,698 (2.6%)	1,000 (2.9%)	745 (2.7%)	0.2	698 (2.0%)	1,000 (3.4%)	*-* 1.4
Divorced	1,110 (1.8%)	3,492 (5.4%)	652 (1.9%)	458 (1.7%)	0.2	1,988 (5.6%)	1,504 (5.1%)	0.5
Widowed	2,931 (4.8%)	1,852 (2.9%)	1,620 (4.8%)	1,311 (4.8%)	0.0	1,110 (3.1%)	742 (2.5%)	0.6
Domestic partner	2,505 (4.1%)	5,778 (8.9%)	1,227 (3.6%)	1,278 (4.7%)	*-* 1.1	2,086 (5.9%)	3,692 (12.5%)	*-* 6.6
(Missing)	212 (0.3%)	1,172 (1.8%)	115 (0.3%)	97 (0.4%)	*-* 0.1	408 (1.2%)	764 (2.6%)	*-* 1.4
Education (years), n (%)								
Up to 8	24,930 (40.8%)	6,978 (10.7%)	13,605 (40.1%)	11,325 (41.7%)	*-* 1.6	3,753 (10.6%)	3,225 (10.9%)	*-* 0.3
9–15	30,719 (50.3%)	44,102 (67.9%)	16,964 (50.0%)	13,755 (50.7%)	*-* 0.7	23,441 (66.4%)	20,661 (69.7%)	*-* 3.3
16 or more	5,367 (8.8%)	13,835 (21.3%)	3,331 (9.8%)	2,036 (7.5%)	2.3	8,092 (22.9%)	5,743 (19.4%)	3.5
(Missing)	30 (0.0%)	8 (0.0%)	10 (< 0.1%)	20 (0.1%)	0.0	1 (< 0.1%)	7 (< 0.1%)	0.0
Employment status, n (%)								
Employed for employer	10,948 (17.9%)	26,811 (41.3%)	6,334 (18.7%)	4,614 (17.0%)	1.7	15,297 (43.4%)	11,514 (38.9%)	4.5
Self-employed	19,120 (31.3%)	10,036 (15.5%)	10,612 (31.3%)	8,508 (31.4%)	*-* 0.1	5,197 (14.7%)	4,839 (16.3%)	*-* 1.6
Retired	3,091 (5.1%)	5,621 (8.7%)	1978 (5.8%)	1,113 (4.1%)	1.7	4,061 (11.5%)	1,560 (5.3%)	6.2
Student	3,688 (6.0%)	3,995 (6.2%)	2,057 (6.1%)	1,631 (6.0%)	0.1	1,733 (4.9%)	2,262 (7.6%)	*-* 2.7
Homemaker	14,154 (23.2%)	6,429 (9.9%)	7,367 (21.7%)	6,787 (25.0%)	*-* 3.3	2,973 (8.4%)	3,456 (11.7%)	*-* 3.3
Unemployed and looking	8,180 (13.4%)	6,244 (9.6%)	4,597 (13.6%)	3,583 (13.2%)	**0.4**	2,654 (7.5%)	3,590 (12.1%)	*-* **4.6**
None of these/Other	1,770 (2.9%)	5,202 (8.0%)	916 (2.7%)	854 (3.1%)	*-* 0.4	3,252 (9.2%)	1,950 (6.6%)	2.6
(Missing)	95 (0.2%)	585 (0.9%)	49 (0.1%)	46 (0.2%)	*-* 0.1	120 (0.3%)	465 (1.6%)	*-* 1.3
Urbanicity status, n (%)								
A rural area or on a farm	20,844 (34.1%)	6,991 (10.8%)	11,645 (34.3%)	9,199 (33.9%)	0.4	3,870 (11.0%)	3,121 (10.5%)	0.5
A small town or village	21,780 (35.7%)	25,304 (39.0%)	12,155 (35.8%)	9,625 (35.5%)	0.3	13,874 (39.3%)	11,430 (38.6%)	0.7
A large city	12,463 (20.4%)	22,103 (34.0%)	6,967 (20.5%)	5,496 (20.3%)	0.2	11,599 (32.9%)	10,504 (35.4%)	*-* 2.5
A suburb of a large city	5,903 (9.7%)	9,574 (14.7%)	3,109 (9.2%)	2,794 (10.3%)	*-* **1.1**	5,779 (16.4%)	3,795 (12.8%)	**3.6**
(Missing)	56 (0.1%)	951 (1.5%)	34 (0.1%)	22 (0.1%)	0.0	165 (0.5%)	786 (2.7%)	*-* 2.2

^*^d difference between retained and not-retained percentages (not number of participants). All reported statistics are unweighted to provide a clear view of the characteristics of the sample. Countries excluded from the analysis: Australia, Germany, Hong Kong, Spain, Sweden, United Kingdom, and United States. The face-to-face interview sample from Nigeria excluded (*n* = 550)

Looking for unequal differences between the retained and not-retained percentages within each mode may provide some evidence of a potential mode effect, where participation in Wave 2 is associated with a particular group that differentially participates, possibly because of the mode they used to take the survey. Differences that stood out more sharply, which may point to a potential mode effect, are bolded in Table 5.

## Strengths and limitations

Wave 2 and future waves of the GFS have several strengths and limitations that researchers should be aware of when interpreting results. Similar to Wave 1, a notable strength is that these data represent a large and diverse sample weighted to be approximately nationally representative of the general adult (18 +) population in each of the 22 countries and one territory, resulting in broad population coverage. Researchers might explore opportunities to build on the geographic and cultural reach of the GFS to include other contexts and communities that tend to be underrepresented in research on well-being, ideally supported by funding mechanisms aiming to promote a more flourishing global population. A notable strength of the second data release compared to the first is the inclusion of mainland China, which expands the global representation of the GFS. The use of the mid-year retention survey between Waves 1 and 2 provided a valuable mid-wave contact point to help reduce dropout rates in many countries. A methodological strength of the approach to collecting longitudinal data in the GFS is the commitment to ensuring respondents use the same mode to complete each survey, which helps maintain within-participant comparability of responses over time without introducing possible mode effects (although other mode effects may potentially limit comparability between participants).

Limitations of the GFS, starting with the use of mid-year data onwards, include difficulties with attrition/participant dropout and differential time lags between data collections by country (though most are approximately one year). The design of the GFS allows for non-monotonic participation, such that a participant who has not responded to the Wave 2 Annual Survey would be invited to respond to the Wave 3 Annual Survey. The retention rate from Wave 1 to 2 varied widely by country, with a low of 23.5% in Hong Kong; however, the overall retention rate from Wave 1 to 2 was above 60% (and above 75% in China, Sweden, and the United States), demonstrating quite considerable success in retaining participants in such a large-scale multinational study. The differential time lags between data collections across countries may limit the comparability of estimates across countries. Longitudinal statistical analyses could also incorporate individual-level time lags. The relevance of differential time lags might also vary across outcomes. While a one-year lag might generally be helpful when studying certain well-being indicators, a shorter or longer lag may be better for certain construct assessments. A similar sampling scheme is planned for future waves of data collection. This makes these limitations an inherent component of the GFS data.

## Wave 3 retention and future waves

Wave 3 data were collected using the same follow-up protocol as described above in the *Wave 2 data collection* section. The overall retention at Wave 3 was 53.6%, ranging from 12.9% in Hong Kong to 85.3% in China, and the retention rates for all countries are reported in Table 6. A major advantage of the follow-up strategy of recontacting all Wave 1 respondents was the participation of those who did not respond in Wave 2 but did respond and participate in Wave 3 (N = 14,802), approximately 7% of the sample. A total of 96,684 people participated in all three waves (~46.5% retention), which can be used for complete-case analyses.

**Table 6:** Retention rates for annual wave 3 data collection

	Annual wave 1	Annual wave 3	
Country	N	N	%-Retained
Overall	207,919	111,486	53.6%
China	5,022	4,283	85.3%
Sweden	15,068	10,425	69.2%
Kenya	11,389	7,796	68.5%
Australia	3,844	2,588	67.3%
United States	38,312	25,543	66.7%
Israel	3,669	2,332	63.6%
United Kingdom	5,368	3,172	59.1%
Tanzania	9,075	5,293	58.3%
Egypt	4,729	2,750	58.2%
India	12,765	6,731	52.7%
Japan	20,543	10,783	52.5%
Poland	10,389	5,399	52.0%
Philippines	5,292	2,753	52.0%
Nigeria	6,827	3,250	47.6%
Germany	9.506	4,497	47.3%
Indonesia	6,992	2,704	38.7%
Spain	6,290	2,406	38.3%
Argentina	6,724	2,322	34.5%
Mexico	5.776	1.618	28.0%
Brazil	13,203	3486	26.4%
Turkey	1.473	359	24.4%
South Africa	2.651	606	22.9%
Hong Kong	3.012	390	12.9%

Wave 4 data is currently collected by Gallup, with future waves of data collection contingent on funding and the feasibility of continuing data collection in countries where retention rates are especially low. Plans include continuing the core survey instrument while considering potential expansion to new modules on digital well-being, climate-related stress, or intergenerational flourishing. New country recruitment may also be explored, particularly in underrepresented regions where there is much to learn about human flourishing; however, incorporating new samples into the panel presents significant methodological complexity, including determining which population targets to use. Current plans are to continue similar sampling and follow-up efforts in subsequent waves, along with similar analytic methods to adjust weights as needed over time.

## Potential research applications

With 2 + waves of GFS data collected, there are many potential applications of these data concerning longitudinal analyses. While coordinated efforts are ongoing [24–27], many possible avenues can be explored that we hope will be taken up by interested research teams. For example, epidemiological analyses can examine within-country change in indicators of well-being from Wave 1 to Wave 2, such as shifts in mean scores or the prevalence of low/high scores on a given indicator. The data are also capable of supporting prospective analyses of Wave 1 predicting Wave 2 outcomes, such as whether risk factors (e.g., suffering) or public health resources (e.g., forgiveness) assessed at baseline predict subsequent changes in well-being outcomes [28]. Analyses can be carried out separately by country to better understand the distribution and potential determinants of various aspects of health and well-being (see [18] for more on country-specific perspectives). Beyond these uses, GFS data may be linked to external country-level (e.g., GDP) or country-specific sources (e.g., social welfare uptake) to explore how sociocultural dynamics might shape well-being [29].

## Conclusion

The GFS is beginning to mature into a central resource for advancing global well-being science. With Waves 2 and 3 completed and longitudinal data now available, researchers can now examine within-person changes in flourishing, and their predictors, across diverse cultural contexts. Future waves of data collection will employ similar sampling methodology and weighting procedures, which will help support the comparability of analyses over time. The scale, methodological strengths, and open science approach of the GFS position it as a unique and important resource for developing a more inclusive and representative understanding of human flourishing and reshaping the global landscape of policy-relevant research on this important topic.

## Supplementary Information

Below is the link to the electronic supplementary material.

Supplementary file1 (DOCX 91 KB)

Supplementary file2 (XLSX 58 KB)
