# Supplementary material for: Cohort update: waves 2 and 3 of the global flourishing study
Source: Eur J Epidemiol. 2026 Jul 16;41(7):893–905. doi: 10.1007/s10654-026-01440-z (PMC13572324; doi:10.1007/s10654-026-01440-z)
Supplement: Supplementary file 1 [file 10654_2026_1440_MOESM1_ESM.docx]

**Online Supplement**

Cohort update: Waves 2 and 3 of the Global Flourishing Study

The online supplement is divided into three major sections. Part A provides additional technical details on the weighting procedures. Part B provides the mid-year survey questions. And Part C provides supplemental tables about response rates and data collection timing.

*Table of Contents*

Part A. Sampling Design and Weighting (pp. 1-5)

*Table A1. Global Flourishing Study sampling summary across countries for Wave 1 (p.2)*

Part B. Mid-year Survey Questions & Variable Map across Waves. (pp. 6-13)

*Table B1.* *Variable map of the availability of each item across waves of data collection (p.9)*

Part C. Supplemental Tables (pp. 14-16)

*Table C1. Date ranges of data collection for the Annual Wave 1, Mid-year Retention, and Annual Wave 2 surveys of the Global Flourishing Study. (p. 14)*

*Table C2. Differences in days between Wave 2 and Wave 1 Global Flourishing Study Annual Survey administration date by country (in days). (p. 15)*

*Table C3. Annual Wave 2 survey retention rates by mode of survey completion. (p.16 )*

**Part A. Weights**

***Sampling Design Description***

Much of the following information is taken directly from the Wave 1 cohort description paper, but is replicated here to provide consistency of reporting with the addition of mainland China. The following is adapted from [16].

Three major sampling frames were used for recruitment in the GFS, namely a probability-based sample, a non-probability-based sample, or a combination of the two [17]. Table 1 summarizes the sampling design within each country. A probability-based sampling approach was used in Egypt, India, Indonesia, Israel, Kenya, Nigeria, the Philippines, South Africa, Tanzania, Turkey, and the United States. A non-probability-based sample was recruited in some countries to supplement probability samples so that adequate coverage of population subgroups (i.e., sex, age, region) was achieved. Recruitment and empanelment for Wave 1 occurred between April 2022 and December 2023 [17].

Data from Hong Kong (S.A.R. of China) became available in the first wave of data collection. Data from mainland China were not included in the first data release due to fieldwork delays. The first wave of fieldwork in mainland China began in February 2024, and a second wave occurred in November-December 2024. All Wave 1 and 2 data from mainland China are part of the second dataset released in March 2025.

| **Table A1**.  *Global Flourishing Study sampling summary across countries for Wave 1*. | | | | |
| --- | --- | --- | --- | --- |
| Country | Sampling Design | Dates of Collection | # Participants | Total Sample Size |
| Argentina | Probability | 11/29/22 - 11/30/23 | 3879 | 6724 |
|  | Non-probability | 04/11/23 - 11/14/23 | 2845 |  |
| Australia | Probability | 03/21/22 - 09/26/23 | 3377 | 3844 |
|  | Non-probability | 04/12/23 - 09/29/23 | 467 |  |
| Brazil | Probability | 11/23/22 - 11/26/23 | 3793 | 13204 |
|  | Non-probability | 04/11/23 - 11/07/23 | 9411 |  |
| China | Non-probability | 03/04/24 - 12/03/24 | 5022 | 5022 |
| Egypt | Probability | 03/09/23 - 09/17/23 | 4729 | 4729 |
| Germany | Probability | 08/11/22 - 11/16/23 | 4480 | 9506 |
|  | Non-probability | 06/12/23 - 08/27/23 | 5026 |  |
| Hong Kong  (S.A.R. of China) | Non-probability | 10/10/23 - 11/24/23 | 3012 | 3012 |
| India | Probability | 04/25/23 - 12/08/23 | 12765 | 12765 |
| Indonesia | Probability | 11/07/22 - 10/27/23 | 6992 | 6992 |
| Israel | Probability | 11/07/22 - 11/23/23 | 3669 | 3669 |
| Japan | Non-probability | 12/13/22 - 06/30/23 | 20543 | 20543 |
| Kenya | Probability | 04/13/23 - 11/21/23 | 11389 | 11389 |
| Mexico | Probability | 10/29/22 - 12/13/23 | 2672 | 5776 |
|  | Non-probability | 05/09/23 - 11/20/23 | 3104 |  |
| Nigeria | Probability | 05/16/23 - 11/17/23 | 6827 | 6827 |
| Philippines | Probability | 04/04/23 - 01/05/24 | 5292 | 5292 |
| Poland | Probability | 12/14/22 - 10/13/23 | 8444 | 10389 |
|  | Non-probability | 06/10/23 - 10/16/23 | 1945 |  |
| South Africa | Probability | 02/26/23 - 12/08/23 | 2651 | 2651 |
| Spain | Probability | 08/17/22 - 11/08/23 | 2309 | 6290 |
|  | Non-probability | 06/22/23 - 08/30/23 | 3981 |  |
| Sweden | Non-probability | 01/16/23 - 02/22/23 | 15068 | 15068 |
| Tanzania | Probability | 02/17/23 - 11/30/23 | 9075 | 9075 |
| Turkey | Probability | 04/15/23 - 01/05/24 | 1473 | 1473 |
| United Kingdom | Probability | 04/26/22 - 11/20/23 | 2341 | 5368 |
|  | Non-probability | 06/12/23 - 08/30/23 | 3027 |  |
| United States | Probability | 08/04/22 - 04/04/23 | 38312 | 38312 |
| *Note*. Probability-based samples included a mix of face-to-face, telephone, and online data collection modes. | | | | |

***Wave 2 Weights Description***

The GFS Year 2 dataset includes five different sets of weights, each suitable for different analysis needs. The decision of which weight to use depends on (1) the target population for which the results are meant to be generalized and (2) the particular period relevant to the analysis. The following weights can be used to estimate approximately nationally representative statistics, provided all cases within the country that have a weight are used. These weights do not account for possible item-level missing data, which must be accounted for before a weighted analysis can be used to estimate approximately nationally representative statistics. The five sets of weights are as follows:

- ANNUAL_WEIGHT_R2: The rectangular weight for all GFS panel members, with demographic information updated based on Year 2 responses, if available. Use this weight to obtain approximately nationally representative statistics for Year 2 based on all GFS panel members.
- ANNUAL_WEIGHT_C2: A cross-sectional weight variable for GFS panel members who completed the Year 2 survey when analyzing the subset of respondents observed in Year 2. Use this weight to estimate approximately nationally representative statistics for Year 2 respondents only. This weight is missing (NA) for respondents who did not participate in Year 2.
- ANNUAL_WEIGHT_L2: The longitudinal weights for GFS panel members who completed both the Year 1 and Year 2 surveys. Use this weight for longitudinal analyses comparing Year 1 and Year 2 under complete case analysis.
- RETENTION_WEIGHT_C: A weight variable for the subset of GFS panel members who participated in the Year 1 mid-year survey. Use this weight for longitudinal analyses examining relationships between Year 1 and Year 1 mid-year variables, or for analyses of variables measured in the Year 1 mid-year survey alone, after subsetting to cases with a valid weight. This weight is missing (NA) for respondents who did not participate in the Year 1 mid-year survey.
- RETENTION_WEIGHT_L: The longitudinal weights for GFS panel members who completed the Year 1 survey, the Year 1 mid-year survey and the Year 2 survey. Use this weight for longitudinal analyses across all the existing three time points.

***Wave 2 Weights Calculation***

For adjusting weights designed for a subset of participants (e.g., analyses restricted to participants who completed a specific wave), a logistic regression-based adjustment is applied to account for nonresponse after the Wave 1 annual survey [18]. The logistic regression model predicts the likelihood of response, with the dependent variable indicating whether a GFS panel member completed the selected survey(s) (coded as 1) or did not complete them (coded as 0). The independent variables in the model include age, gender, education, region, marital status, and employment status. Country-specific education categories were also used, but were typically aggregated into three or four groups to align with the categories used for raking. A similar approach was taken with the region variable, where the same categories used for post-stratification adjustment are included in the logistic regression. Where available, all variables except age and gender were updated based on the most recent survey responses available; age was updated for all cases using the respondent’s date of birth or an estimated birth date based on their reported age in Wave 1. Otherwise, Wave 1 values were used.

If any values remained missing after incorporating valid responses from Wave 1 and Wave 2, they were imputed using the unweighted mode of the sample. These imputed values were used for a small proportion of the sample, and solely for weighting purposes. The imputed values are not included in the released dataset. The proportion of cases with missing values on any demographic characteristic included in the weighing adjustments ranged from 0.04% of cases in China to 3.26% in Brazil.

The predicted probabilities from the logistic regression model were then used to compute adjustment weights. These weights reflect the inverse of the estimated probability of responding. Respondents with a higher probability of response receive a lower weight, while those with a lower probability receive a higher weight. This adjustment corrects for the over-representation of individuals who were more likely to respond and the under-representation of those who were less likely to respond. There was no trimming of weights at this stage.

The above describes the additional procedures needed to adjust the Wave 1 weights for analyses that use only a subset of the full sample. Another type of weight is used involving all empaneled participants regardless of nonresponse after Wave 1, but the weight still accounts for selection nonresponse into the panel (i.e., people more or less likely to participate in the panel at all) [16-17]. For this "all cases" weight, the following calibration and trimming adjustments were used to adjust the base weight from Wave 1, but without the logistic regression adjustment for nonresponse, because the full sample was utilized.

The next step involved calibrating the nonresponse-adjusted sampling weights. The weights were calibrated using a raking procedure to align with known population totals. The characteristics used for raking typically included marginal or joint distributions of age, gender, region, education, marital status, employment status, and race/ethnicity, where possible. The selection of characteristics varied by country based on data availability. The selected targets and characteristics were consistent with those used in Wave 1 annual weighting [16].

For the GFS, the same individuals (or a subset of the same individuals) are followed over time. Keeping the same target ensures their weights remain comparable across years. A stable weighting target ensures that variations in responses are attributable to actual differences in the surveyed population rather than shifts in demographic benchmarks. If different targets are used across years, it is possible that changes in weighted estimates might be due to adjustments in the weighting process rather than changes in population dynamics. A stable population target allows researchers to analyze trends more easily. In cases where certain categories contain zero or very few respondents, they are merged with another category that shares similar characteristics.

Wave 3 weights were calculated using the same methods.

**Part B. Mid-year Survey Questions & Variable Map across Waves.**

**TIME MEDIA**

On average, how much time do you spend each day using social media platforms, such as [insert country-specific social media platforms]? (Interviewer: If respondent says they do not use social media, code as "None")

|  | CIRCLE ONE RESPONSE: |
| --- | --- |
| None | 1 |
| Less than 30 minutes | 2 |
| 30 minutes to less than an hour | 3 |
| 1 to 2 hours | 4 |
| More than 2 hours | 5 |
| (DK) | 98 |
| (Refused) | 99 |

**ENGAGE ARTS**

How often do you engage in an arts or cultural activity, such as singing, painting, playing a musical instrument, drawing, dancing, creative writing, photography, or visiting a museum, theatre, or concert hall? Please think of all the times you engage in these activities, whether it is for enjoyment or you receive payment in return. Is it…?

|  | CIRCLE ONE RESPONSE: |
| --- | --- |
| More than once a week | 1 |
| Once a week | 2 |
| One to three times a month | 3 |
| A few times a year | 4 |
| Never | 5 |
| (DK) | 98 |
| (Refused) | 99 |

**BEAUTY**

Do you regularly experience things that you consider beautiful? This may include physical beauty or abstract beauty like that found in music, art, or nature.

| Yes | No | (DK) | (Refused) |
| --- | --- | --- | --- |
| 1 | 2 | 98 | 99 |

**ACHIEVING**

Are you happy with what you are achieving in life?

| Yes | No | (DK) | (Refused) |
| --- | --- | --- | --- |
| 1 | 2 | 98 | 99 |

**FOOD INSECURE**

In the last 12 months, how frequently did your household run out of food because of a lack of money or other resources? Often, sometimes, or never?

|  | CIRCLE ONE RESPONSE: |
| --- | --- |
| Often | 1 |
| Sometimes | 2 |
| Never | 3 |
| (DK) | 98 |
| (Refused) | 99 |

**NATURE & DILIGENT**

Please imagine a ladder with steps numbered from zero at the bottom to ten at the top. The top of the ladder at ten means you "strongly agree" and the bottom of the ladder at zero means you "strongly disagree." Please rate your level of agreement with the following statements. You can answer any number between 0 and 10; for example, 0, 1, 2, 3, 4, 5, 6, 7, 8, 9, or 10. (Read Items)

|  | | Strongly disagree |  |  |  |  |  |  |  |  |  | Strongly agree | (DK) | (Refused) |
| --- | --- | --- | --- | --- | --- | --- | --- | --- | --- | --- | --- | --- | --- | --- |
| NATURE | You feel very connected to nature | 0 | 1 | 2 | 3 | 4 | 5 | 6 | 7 | 8 | 9 | 10 | 98 | 99 |
| DILIGENT | You are diligent | 0 | 1 | 2 | 3 | 4 | 5 | 6 | 7 | 8 | 9 | 10 | 98 | 99 |

**MIND FOCUSED**

Still thinking about the ladder, with the top of the ladder at ten being "always" and the bottom of the ladder at zero being "never," how often does the following statement apply to you? Your mind is focused on what you do. (Interviewer: If necessary, say: "You can use any number between 0 and 10.")

| Never |  |  |  |  |  |  |  |  |  | Always | (DK) | (Refused) |
| --- | --- | --- | --- | --- | --- | --- | --- | --- | --- | --- | --- | --- |
| 0 | 1 | 2 | 3 | 4 | 5 | 6 | 7 | 8 | 9 | 10 | 98 | 99 |

**VALUE RATINGS**

Now, please think about a ladder with the top of the ladder at ten being "extremely important" and the bottom of the ladder at zero being "not at all important." How important are each of the following to you? You can use any number between 0 and 10.

| Domain | | Not at all important |  |  |  |  |  |  |  |  |  | Extremely important | (DK) | (Refused) |
| --- | --- | --- | --- | --- | --- | --- | --- | --- | --- | --- | --- | --- | --- | --- |
| HAPPY_IMPORT | Being happy | 0 | 1 | 2 | 3 | 4 | 5 | 6 | 7 | 8 | 9 | 10 | 98 | 99 |
| HEALTHY | Being healthy | 0 | 1 | 2 | 3 | 4 | 5 | 6 | 7 | 8 | 9 | 10 | 98 | 99 |
| MEANINGFUL | Having a meaningful life | 0 | 1 | 2 | 3 | 4 | 5 | 6 | 7 | 8 | 9 | 10 | 98 | 99 |
| GOOD_PERSON | Being a good person | 0 | 1 | 2 | 3 | 4 | 5 | 6 | 7 | 8 | 9 | 10 | 98 | 99 |
| GOOD_RELATION | Having good relationships | 0 | 1 | 2 | 3 | 4 | 5 | 6 | 7 | 8 | 9 | 10 | 98 | 99 |
| REL_LIFE | Having a good religious or spiritual life | 0 | 1 | 2 | 3 | 4 | 5 | 6 | 7 | 8 | 9 | 10 | 98 | 99 |
| MONEY | Having plenty of money to have what you want | 0 | 1 | 2 | 3 | 4 | 5 | 6 | 7 | 8 | 9 | 10 | 98 | 99 |

| **Table B1.**  *Variable map of the availability of each item across waves of data collection.* | | | | | |
| --- | --- | --- | --- | --- | --- |
| **VARIABLE** | **YEAR 1** | **RETENTION** | **YEAR 2** | **YEAR 3** | **NOTES** |
| ID | X | X | X | X |  |
| COUNTRY | X | X | X | X |  |
| WAVE | X |  |  |  |  |
| MODE_RECRUIT | X |  |  |  |  |
| MODE_ANNUAL | X |  |  |  |  |
| RECRUIT_TYPE | X |  |  |  |  |
| DOI_RECRUIT | X |  |  |  | DOI, date of interview |
| DOI_ANNUAL | X |  |  |  |  |
| DOI_RETENTION |  | X |  |  |  |
| DOI_ANNUAL_2 |  |  | X |  |  |
| DOI_ANNUAL_3 |  |  |  | X |  |
| ABUSED | X |  |  |  |  |
| AFTER_DEATH | X |  | X | X |  |
| AGE | X | X | X | X |  |
| APPROVE_GOVT | X |  | X | X |  |
| ATTEND_SVCS | X |  | X | X |  |
| BELIEVE_GOD | X |  | X | X |  |
| BELONGING | X |  | X | X |  |
| BODILY_PAIN | X |  | X | X |  |
| BORN_COUNTRY | X |  |  |  |  |
| CAPABLE | X |  | X | X |  |
| CIGARETTES | X |  | X | X |  |
| CLOSE_TO | X |  | X | X |  |
| CNTRY_REL_BUD | X |  |  |  |  |
| CNTRY_REL_CHI | X |  |  |  |  |
| CNTRY_REL_CHR | X |  |  |  |  |
| CNTRY_REL_HIN | X |  |  |  |  |
| CNTRY_REL_ISL | X |  |  |  |  |
| CNTRY_REL_JUD | X |  |  |  |  |
| CNTRY_REL_SHI | X |  |  |  |  |
| COMFORT_REL | X |  | X | X |  |
| CONNECTED_REL | X |  | X | X |  |
| CONTENT | X |  | X | X |  |
| CONTROL_WORRY | X |  | X | X |  |
| COVID_DEATH | X |  |  |  |  |
| CRITICAL | X |  | X | X |  |
| DAYS_EXERCISE | X |  | X | X |  |
| DEPRESSED | X |  | X | X |  |
| DISCRIMINATED | X |  | X | X |  |
| DONATED | X |  | X | X |  |
| DRINKS | X |  | X | X |  |
| EDUCATION | X |  |  |  |  |
| EDUCATION_3 | X |  | X | X |  |
| EMPLOYMENT | X |  | X | X |  |
| EXPECT_GOOD | X |  | X | X |  |
| EXPENSES | X |  | X | X |  |
| FATHER_LOVED | X |  |  |  |  |
| FATHER_RELATN | X |  |  |  |  |
| FEEL_ANXIOUS | X |  | X | X |  |
| FORGIVE | X |  | X | X |  |
| FREEDOM | X |  | X | X |  |
| GENDER | X |  |  |  |  |
| GIVE_UP | X |  | X | X |  |
| GOD_PUNISH | X |  | X | X |  |
| GRATEFUL | X |  | X | X |  |
| GROUP_NOT_REL | X |  | X | X |  |
| HAPPY | X |  | X | X |  |
| HEALTH_GROWUP | X |  |  |  |  |
| HEALTH_PROB | X |  | X | X |  |
| HELP_STRANGER | X |  | X | X |  |
| HOPE_FUTURE | X |  | X | X |  |
| INCOME | X |  | X | X |  |
| INCOME_12YRS | X |  |  |  |  |
| INCOME_DIFF | X |  |  |  |  |
| INCOME_FEELINGS | X |  | X | X |  |
| INTEREST | X |  | X | X |  |
| LIFE_APPROACH | X |  | X | X |  |
| LIFE_BALANCE | X |  | X | X |  |
| LIFE_PURPOSE | X |  | X | X |  |
| LIFE_SAT | X |  | X | X |  |
| LONELY | X |  | X | X |  |
| LOVED_BY_GOD | X |  | X | X |  |
| MARITAL_STATUS | X |  | X | X |  |
| MENTAL_HEALTH | X |  | X | X |  |
| MOTHER_LOVED | X |  |  |  |  |
| MOTHER_RELATN | X |  |  |  |  |
| NUM_CHILDREN | X |  | X | X |  |
| NUM_HOUSEHOLD | X |  | X | X |  |
| OBEY_LAW | X |  |  |  |  |
| OUTSIDER | X |  |  |  |  |
| OWN_RENT_HOME | X |  | X | X |  |
| PARENTS_12YRS | X |  |  |  |  |
| PEACE | X |  | X | X |  |
| PEOPLE_HELP | X |  | X | X |  |
| PHYSICAL_HLTH | X |  | X | X |  |
| POLITICAL_ID | X |  | X | X |  |
| PRAY_MEDITATE | X |  | X | X |  |
| PROMOTE_GOOD | X |  | X | X |  |
| REGION1 | X |  | X | X |  |
| REGION2 | X |  | X | X |  |
| REGION3 | X |  | X | X |  |
| REL_EXPERIENC | X |  | X | X |  |
| REL_IMPORTANT | X |  |  |  |  |
| REL1 | X |  |  |  |  |
| REL2 | X |  | X | X |  |
| REL3 | X |  | X | X |  |
| REL4 | X |  | X | X |  |
| REL5 | X |  | X | X |  |
| REL6 | X |  | X | X |  |
| REL7 | X |  | X | X |  |
| REL8 | X |  | X | X |  |
| REL9 | X |  | X | X |  |
| SACRED_TEXTS | X |  | X | X |  |
| SAT_LIVE | X |  | X | X |  |
| SAT_RELATNSHP | X |  | X | X |  |
| SAY_IN_GOVT | X |  | X | X |  |
| SELFID1 | X |  |  |  |  |
| SELFID2 | X |  |  |  |  |
| SHOW_LOVE | X |  | X | X |  |
| SUFFERING | X |  | X | X |  |
| SVCS_12YRS | X |  |  |  |  |
| SVCS_FATHER | X |  |  |  |  |
| SVCS_MOTHER | X |  |  |  |  |
| TEACHINGS_1 | X |  |  |  |  |
| TEACHINGS_2 | X |  |  |  |  |
| TEACHINGS_3 | X |  |  |  |  |
| TEACHINGS_4 | X |  |  |  |  |
| TEACHINGS_5 | X |  |  |  |  |
| TEACHINGS_6 | X |  |  |  |  |
| TEACHINGS_7 | X |  |  |  |  |
| TEACHINGS_8 | X |  |  |  |  |
| TEACHINGS_9 | X |  |  |  |  |
| TEACHINGS_10 | X |  |  |  |  |
| TEACHINGS_11 | X |  |  |  |  |
| TEACHINGS_12 | X |  |  |  |  |
| TEACHINGS_13 | X |  |  |  |  |
| TEACHINGS_14 | X |  |  |  |  |
| TEACHINGS_15 | X |  |  |  |  |
| TELL_BELIEFS | X |  | X | X |  |
| THREAT_LIFE | X |  | X | X |  |
| TRAITS1 | X |  |  |  |  |
| TRAITS2 | X |  |  |  |  |
| TRAITS3 | X |  |  |  |  |
| TRAITS4 | X |  |  |  |  |
| TRAITS5 | X |  |  |  |  |
| TRAITS6 | X |  |  |  |  |
| TRAITS7 | X |  |  |  |  |
| TRAITS8 | X |  |  |  |  |
| TRAITS9 | X |  |  |  |  |
| TRAITS10 | X |  |  |  |  |
| TRUST_PEOPLE | X |  | X | X |  |
| URBAN_RURAL | X |  | X | X |  |
| VOLUNTEERED | X |  | X | X |  |
| WB_FIVEYRS | X |  | X | X |  |
| WB_TODAY | X |  | X | X |  |
| WORRY_SAFETY | X |  | X | X |  |
| WORTHWHILE | X |  | X | X |  |
| ANNUAL_WEIGHT_C1 | X |  |  |  |  |
| ANNUAL_WEIGHT_C2 |  |  | X |  | Year 2 cross-section (completed Wave 2) |
| ANNUAL_WEIGHT_L2 |  |  | X |  | Year 1 & 2 longitudinal (same as above, will differ in subsequent years) |
| ANNUAL_WEIGHT_R2 |  |  | X |  | All records (regardless of participation in year 2) |
| ANNUAL_WEIGHT_C3 |  |  |  | X | Year 3 cross-section (completed Wave 3) |
| ANNUAL_WEIGHT_L3 |  |  |  | X | Year 1, 2, & 3 longitudinal (complete all waves) |
| ANNUAL_WEIGHT_R3 |  |  |  | X | All records (regardless of participation in year 2 or year 3) |
| RETENTION_WEIGHT_C |  | X |  |  | Retention cross-section (completed year 1 & retention) |
| RETENTION_WEIGHT_L |  | X |  |  | Retention longitudinal (completed year 1 & retention & year 2) |
| STRATA | X |  |  |  |  |
| PSU | X |  |  |  |  |
| FULL_PARTIAL | X | X | X | X |  |
| STUDY_YEAR | X | X | X | X |  |
| MODE | X | X | X | X |  |
| TIME_MEDIA |  | X |  |  |  |
| ENGAGE_ARTS |  | X |  |  |  |
| BEAUTY |  | X |  |  |  |
| ACHIEVING |  | X |  |  |  |
| FOOD_INSECURE |  | X |  |  |  |
| NATURE |  | X |  |  |  |
| DILIGENT |  | X |  |  |  |
| MIND_FOCUSED |  | X |  |  |  |
| HAPPY_IMPORT |  | X |  |  |  |
| HEALTHY |  | X |  |  |  |
| MEANINGFUL |  | X |  |  |  |
| GOOD_PERSON |  | X |  |  |  |
| GOOD_RELATION |  | X |  |  |  |
| REL_LIFE |  | X |  |  |  |
| GF_MONEY |  | X |  |  |  |

**Part C. Supplemental Tables**

| **Table C1**.  *Date ranges of data collection for the Annual Wave 1, Mid-year Retention, and Annual Wave 2 surveys of the Global Flourishing Study.* | | | |
| --- | --- | --- | --- |
| Country | Wave 1 | Mid-year Retention | Wave 2 |
| Argentina | 2023-01-31 / 2023-11-30 | 2024-02-16 / 2024-12-30 | 2024-07-15 / 2024-12-30 |
| Australia | 2022-03-29 / 2023-09-29 | 2023-11-15 / 2024-04-21 | 2024-05-08 / 2024-09-25 |
| Brazil | 2023-01-31 / 2023-11-26 | 2024-03-04 / 2024-12-27 | 2024-07-23 / 2024-12-29 |
| China | 2024-03-04 / 2024-04-12 | 2024-10-22 / 2024-12-13 | 2024-10-22 / 2024-12-13 |
| Egypt | 2023-03-29 / 2023-09-17 | 2024-03-11 / 2024-04-29 | 2024-07-30 / 2024-11-06 |
| Germany | 2022-10-17 / 2023-11-16 | 2023-11-27 / 2024-09-20 | 2024-05-16 / 2024-11-21 |
| Hong Kong | 2023-10-10 / 2023-11-24 | 2024-07-15 / 2024-11-15 | 2024-07-15 / 2024-11-15 |
| India | 2023-05-06 / 2023-12-08 | 2024-02-22 / 2024-05-20 | 2024-07-23 / 2024-12-31 |
| Indonesia | 2022-11-21 / 2023-10-27 | 2024-02-15 / 2024-05-14 | 2024-06-19 / 2024-12-29 |
| Israel | 2022-11-17 / 2023-11-23 | 2024-06-17 / 2024-11-21 | 2024-06-17 / 2024-11-21 |
| Japan | 2022-12-13 / 2023-06-30 | 2024-03-01 / 2024-06-26 | 2024-03-01 / 2024-06-26 |
| Kenya | 2023-05-25 / 2023-11-21 | 2024-02-17 / 2024-05-23 | 2024-07-23 / 2024-12-29 |
| Mexico | 2022-11-17 / 2023-12-13 | 2024-02-16 / 2024-12-30 | 2024-07-23 / 2024-12-30 |
| Nigeria | 2023-05-25 / 2023-11-17 | 2024-02-14 / 2024-11-05 | 2024-07-25 / 2024-12-19 |
| Philippines | 2023-04-16 / 2024-01-05 | 2024-02-13 / 2024-05-15 | 2024-07-20 / 2024-12-28 |
| Poland | 2023-01-27 / 2023-10-16 | 2024-02-17 / 2024-05-12 | 2024-06-25 / 2024-12-31 |
| South Africa | 2023-04-12 / 2023-12-08 | 2024-02-12 / 2024-06-05 | 2024-07-22 / 2024-12-29 |
| Spain | 2022-11-07 / 2023-11-08 | 2023-11-24 / 2024-12-22 | 2024-05-16 / 2024-12-22 |
| Sweden | 2023-01-16 / 2023-03-23 | 2024-02-12 / 2024-03-18 | 2024-02-12 / 2024-03-18 |
| Tanzania | 2023-03-16 / 2023-11-30 | 2024-02-15 / 2024-05-24 | 2024-07-22 / 2024-12-21 |
| Turkey | 2023-05-10 / 2024-01-05 | 2024-02-14 / 2024-04-30 | 2024-07-17 / 2024-12-05 |
| United Kingdom | 2022-06-10 / 2023-11-20 | 2023-11-20 / 2024-09-20 | 2024-05-07 / 2024-11-04 |
| United States | 2022-08-04 / 2023-04-04 | 2024-01-22 / 2024-07-30 | 2024-01-22 / 2024-07-30 |
|  |  |  |  |
| Overall | 2022-03-29 / 2024-04-12 | 2023-11-15 / 2024-12-30 | 2024-01-22 / 2024-12-31 |

| **Table C2**.  *Differences in days between Wave 2 and Wave 1 Global Flourishing Study Annual Survey administration date by country* (in days)*.* | | | | | | | |
| --- | --- | --- | --- | --- | --- | --- | --- |
|  |  |  | Difference between Waves 1 and 2  administrations (in days) | | | | |
| Country | Wave 1 Start | Wave 2 Start | Mean (SD) | Minimum | Q1 | Q3 | Maximum |
| Australia | 3/29/22 | 5/8/24 | 565.9 (146.0) | 243 | 450 | 704 | 868 |
| Indonesia | 11/21/22 | 6/19/24 | 550.0 (88.6) | 265 | 529 | 608 | 717 |
| United States | 8/4/22 | 1/22/24 | 483.2 (93.3) | 295 | 360 | 538 | 661 |
| Tanzania | 3/16/23 | 7/22/24 | 451.3 (63.4) | 248 | 404 | 500 | 618 |
| Kenya | 5/25/23 | 7/23/24 | 450.7 (65.6) | 248 | 419 | 500 | 580 |
| Poland | 1/27/23 | 6/25/24 | 431.6 (50.6) | 295 | 392 | 458 | 688 |
| Egypt | 3/29/23 | 7/30/24 | 420.5 (30.2) | 320 | 405 | 440 | 576 |
| Nigeria | 5/25/23 | 7/25/24 | 414.3 (50.3) | 261 | 383 | 443 | 564 |
| United Kingdom | 6/10/22 | 5/7/24 | 409.2 (111.0) | 201 | 332 | 498 | 876 |
| India | 5/6/23 | 7/23/24 | 406.2 (70.0) | 229 | 355 | 462 | 580 |
| Brazil | 1/31/23 | 7/23/24 | 405.4 (78.6) | 240 | 359 | 438 | 687 |
| Argentina | 1/31/23 | 7/15/24 | 402.6 (94.9) | 243 | 315 | 472 | 673 |
| South Africa | 4/12/23 | 7/22/24 | 401.2 (69.4) | 229 | 356 | 446 | 595 |
| Israel | 11/17/22 | 6/17/24 | 394.0 (102.0) | 210 | 298 | 472 | 713 |
| Mexico | 11/17/22 | 7/23/24 | 394.0 (89.4) | 250 | 323 | 451 | 733 |
| Turkey | 5/10/23 | 7/17/24 | 369.9 (63.8) | 233 | 326 | 416 | 531 |
| Philippines | 4/16/23 | 7/20/24 | 369.5 (70.8) | 229 | 317 | 423 | 581 |
| Sweden | 1/16/23 | 2/12/24 | 367.9 (18.9) | 329 | 353 | 386 | 422 |
| Germany | 10/17/22 | 5/16/24 | 363.2 (57.5) | 216 | 331 | 363 | 684 |
| Japan | 12/13/22 | 3/1/24 | 350.1 (30.5) | 277 | 331 | 376 | 459 |
| Spain | 11/7/22 | 5/16/24 | 348.6 (104.0) | 227 | 274 | 376 | 733 |
| Hong Kong | 10/10/23 | 7/15/24 | 301.9 (19.8) | 274 | 287 | 311 | 386 |
| China | 3/4/24 | 10/22/24 | 239.8 (12.3) | 197 | 233 | 250 | 270 |
|  |  |  |  |  |  |  |  |
| Overall | 2022-03-29 | 2024-01-22 | 417.5 (98.2) | 197 | 344 | 520 | 876 |
| *Note.* Countries are sorted by the average length of lag between Waves 1 and 2 of data collection. | | | | | | | |

| **Table C3.**  *Annual Wave 2 survey retention rates by mode of survey completion.* | | | | | | | | | |
| --- | --- | --- | --- | --- | --- | --- | --- | --- | --- |
|  | Wave 1 | | |  | Wave 2 | | | | |
| Country | Total  *N* | Web  *N* | Phone  *N* |  | Total  *N* | Web  *N* | Web  *%-Retained* | Phone  *N* | Phone  *%-Retained* |
| Overall | 207,919 | 146,323 | 61,046 |  | 128,868 | 94,502 | 64.6 | 33,910 | 55.5 |
|  |  |  |  |  |  |  |  |  |  |
| Argentina | 6,724 | 5,273 | 1,451 |  | 2,932 | 2,158 | 40.9 | 774 | 53.3 |
| Australia | 3,844 | 3,844 | 0 |  | 2,582 | 2,582 | 67.2 | 0 | - |
| Brazil | 13,203 | 11,938 | 1,265 |  | 4,274 | 3,735 | 31.3 | 539 | 42.6 |
| China | 5,022 | 4,178 | 844 |  | 4,544 | 3,778 | 90.4 | 766 | 90.8 |
| Egypt | 4,729 | 221 | 4,508 |  | 3,040 | 107 | 48.4 | 2,933 | 65.1 |
| Germany | 9,506 | 9,506 | 0 |  | 5,529 | 5,529 | 58.2 | 0 | - |
| Hong Kong | 3,012 | 3,012 | 0 |  | 707 | 707 | 23.5 | 0 | - |
| India | 12,765 | 216 | 12,549 |  | 6,374 | 63 | 29.2 | 6,311 | 50.3 |
| Indonesia | 6,992 | 3,372 | 3,620 |  | 2,684 | 1,337 | 39.7 | 1,347 | 37.2 |
| Israel | 3,669 | 926 | 2,743 |  | 2,490 | 246 | 26.6 | 2,244 | 81.8 |
| Japan | 20,543 | 20,001 | 542 |  | 13,972 | 13,680 | 68.4 | 292 | 53.9 |
| Kenya | 11,389 | 1,472 | 9,917 |  | 7,698 | 1,023 | 69.5 | 6,675 | 67.3 |
| Mexico | 5,776 | 4,217 | 1,559 |  | 2,278 | 1,579 | 37.4 | 699 | 44.8 |
| Nigeria* | 6,827 | 668 | 5,609 |  | 3,146 | 392 | 58.7 | 2,298 | 41.0 |
| Philippines | 5,292 | 1,118 | 4,174 |  | 2,682 | 505 | 45.2 | 2,177 | 52.2 |
| Poland | 10,389 | 9,764 | 625 |  | 6,478 | 6,052 | 62.0 | 426 | 68.2 |
| South Africa | 2,651 | 641 | 2,010 |  | 978 | 267 | 41.7 | 711 | 35.4 |
| Spain | 6,290 | 6,290 | 0 |  | 2,924 | 2,924 | 46.5 | 0 | - |
| Sweden | 15,068 | 15,068 | 0 |  | 11,609 | 11,609 | 77.0 | 0 | - |
| Tanzania | 9,075 | 285 | 8,790 |  | 5,583 | 166 | 58.2 | 5,417 | 61.6 |
| Turkey | 1,473 | 633 | 840 |  | 500 | 199 | 31.4 | 301 | 35.8 |
| United Kingdom | 5,368 | 5,368 | 0 |  | 3,619 | 3,619 | 67.4 | 0 | - |
| United States | 38,312 | 38,312 | 0 |  | 32,245 | 32,245 | 84.2 | 0 | - |
| *Note.* *Nigeria sampling includes a 550-participant subsample with face-to-face interviews. Baseline N = 550, Wave 2 N = 456, %-retained = 82.9%. | | | | | | | | | |
